# In the shadow of dissidence: exploring the experiences of descendants of Czechoslovakian dissidents

**DOI:** 10.3389/fpsyg.2024.1310238

**Published:** 2024-02-19

**Authors:** Nikola Doubková, Radek Heissler, Edel Sanders, Marek Preiss

**Affiliations:** ^1^National Institute of Mental Health, Klecany, Czechia; ^2^School of Psychology, University of New York in Prague, Prague, Czechia

**Keywords:** expulsion, interpretative phenomenological analysis, dissidents, intergenerational, 1.5 generation

## Abstract

**Introduction:**

The Communist Party’s reign in Czechoslovakia (1948–1989) saw the persecution of thousands of individuals. The State Security campaign “Asanace” (meaning “sanitation”) was conducted to expel critics of the regime from the country using psychological and physical terror. Although stories of dissidents are frequently presented in public spaces, little is known about the experiences of their children.

**Methods:**

To address this gap, we conducted interpretative phenomenological analyses of semi-structured in-depth interviews with five adult descendants of Czechoslovakian dissidents.

**Results:**

Our analyses revealed that while participants appreciated and were inspired by their parents’ dissident activities, they tend to distance themselves from it in order not to live in their parents’ shadow. Furthermore, for them, the “Asanace” campaign primarily meant emigration, which in turn affected their sense of self and (national) identity dispersion. Consequently, they experienced feelings of being uprooted and different. Furthermore, they faced challenges acculturating. However, they also recognized their resilience as being rooted in their migration experience and the legacy of their parents’ dissidence.

**Discussion:**

By highlighting intergenerational differences and the impact of family legacy on individuals’ strengths and weaknesses, this study contributes to our understanding of the psychological consequences of living in, escaping from and adjusting to life beyond oppressive regimes.

## 1 Introduction

The experience of certain traumatic events can lead to profound universal or specific cognitive or emotional reactions (Dalenberg et al., 2017, 27). Such events might even be significant turning points in history, such as the Holocaust. The impact of these events is not limited to the individuals who directly experienced them, but also can have a transgenerational effect. Studies have shown that in many populations, the psychological effects of the events that parents have lived through can be transmitted to their second (i.e., those born after the event) or even third (i.e., descendants of the second generation) generations (e.g., Burchert et al., 2017; Cohn and Morrison, 2018; Dikyurt, 2023).

Psychological consequences of certain historical events across generations are extensively studied, especially the Holocaust, which refers to the unprecedented systematic persecution and murder of Jews and other minority groups between 1933 and 1945 in Nazi Germany and its occupied territories (Berenbaum, 2023). However, the consequences of other historical events that are less widely recognized or less extreme in their nature should not be overlooked, as they could help one to understand how such historical and potentially traumatic events might affect generations of people and their mental health. This could be explored, for example, via a sample of victims of state oppression during the communist regime in former Czechoslovakia.

The Communist Party of Czechoslovakia gained control over the country in 1948 and remained in control for the next 40 years. During that time, the persecution of thousands of individuals occurred (for details see, e.g., Heimann, 2011; Hauner et al., 2023). During the 1970s and 1980s, the code-named “Asanace” (meaning sanitation or clearance) campaign was organized by the State Security (the secret police) with the aim to expel critics of the regime, political dissidents, and their families from the country. This campaign used tactics such as harassment, torture, intimidation, and other forms of psychological and physical abuse. Most of these dissidents were people actively involved in the Charter 77 initiative criticizing the government for not observing human rights as outlined in the Helsinki Accords of 1975. Signing and spreading the Charter 77 petition was considered a political crime (Gregor, 2006; Koutek, 2006). Data has not been found that indicates precisely how many people were affected by the “Asanace” campaign and how many people were expelled from Czechoslovakia in the end. We know that all 1,882 people who signed Charter 77 were in the crosshairs of State Security; it is estimated that from those, around 280 emigrated. However, the exact number of people involved in the systematic psychological and physical oppression led under the “Asanace” campaign is unknown. Koutek (2006) estimates that 70 people were expelled between 1978 and 1981; the number of affected families is smaller, as more than one family member was often involved in the campaign. Since 1981, however, State Security was instructed to ensure that there would not be an excessive brain drain, which thwarted the original purpose of the campaign (Koutek, 2006).

The case of the Czechoslovakian “Asanace” campaign serves as an example of how expulsion is employed as one of the instruments in political purges within totalitarian regimes, which was common in all the Eastern and Central European countries under the soviet sphere of power. Yet similar procedures can be observed in other totalitarian regimes worldwide, where critics and dissidents, along with their families, are targeted and forcibly removed from the country to suppress opposition and maintain control (Montagnes and Wolton, 2019).

A specific position in studies of psychological consequences of such historical events and their transmission refers to a “1.5 generation” (Suleiman, 2002; Rumbaut, 2004). This denotes the children who were old enough to live through the event themselves but too young to have a mature understanding of what was happening to them or their families (Suleiman, 2002). Moreover, these children are considered dually traumatized, once by their own early exposure, and secondly by intergenerational transmission due to growing up with parents who were also affected by the event (Sossin, 2007). Of course, the age of the child at the time of these historical traumatic experiences plays a crucial role in further development, adaptation, acculturation, identity formation, and overall mental health (Rumbaut, 2004; Kebede, 2010; Tan, 2016; Gindelsky, 2019).

The 1.5 generation can be divided into three distinct groups based on their age when the event occurred (Suleiman, 2002, 283; Rumbaut, 2004): (1) “too young to remember” (infancy to pre-school age), i.e., those who have no memory of the events and are generally very similar to the second generation, (2) “old enough to remember but too young to understand” (primary school children and pre-adolescents), and (3) “old enough to understand but too young to be responsible” (adolescents), whose experiences and adaptive outcomes are deemed to be closer to the first generation, and thus are considered the most vulnerable. These differences also influence the memories and narratives of those who reflect on their experiences years later (Suleiman, 2002).

This study will focus on children in the borderline between the first and the second group mentioned. Around the age of 4 to 5, children gain the ability to make temporal connections, develop the necessary level of awareness for episodic memory, and make connections between past events and the present, i.e., essential requirements for autobiographical memories (Povinelli et al., 1999; Lemmon and Moore, 2001; Brown et al., 2009; Bartels et al., 2021). As Lagattuta and Wellman (2001) suggested, children in preschool years begin to develop a coherent comprehension of life history, mind, and emotion. Trauma exposure during the preschool and early school years can detrimentally affect developmental tasks and challenges children face at this stage, such as self-regulation, the development of coping capacities, the formation of secure attachments, and the ability to relate to peers (Dunn et al., 2017).

Although stories of Czechoslovakian dissidents are frequently presented in public spaces and in research studies, only minimal attention is given to experiences of their children and their points of view. To emphasize the importance of examining their experiences, it should be noted that these are children who were exposed to these potentially traumatic oppressive strategies of the totalitarian regime themselves. Thus, in this study, we focus on subjective lived experiences of children (now adults) who were old enough to remember but too young to understand during that time how the “Asanace” campaign significantly affected their families. We conducted semi-structured interviews with now-adult descendants of Czechoslovakian dissidents applying an interpretative phenomenological approach (Smith et al., 2009) to seek understanding of their complex and previously unexplored experiences as well as their thoughts and ideas about their family history, as well as the impact this has had on their lives (McCormack and Joseph, 2018).

## 2 Materials and methods

### 2.1 Data collection

The participant recruitment began in May 2020 and was concluded in July 2022. Interestingly, there is no definitive list of people affected by the “Asanace” campaign as noted above; only a list of people that historians suspect were affected is publicly available. Nonetheless, we reached out to the people listed with a request to participate in our study. The study, its goals, and the research team were introduced during the first contact. Initial contact with potential participants was established either through email or face-to-face communication. The participants were contacted directly, as most of them are publicly known figures; however, later, a snowball technique was also used. All participants were asked whether they were affected by the “Asanace” campaign according to the State Security documents available to them; only those who were affected were included in the study. Overall, we contacted 49 families and subsequently checked the inclusion criteria. In total, 18 participants (i.e., families) from the first generation met the requirements and were willing and able to participate. Their descendants were then reached by three means: (1) either a contact was given to us with the consent of the offspring, (2) the offspring contacted us directly, or (3) some descendants were approached through the snowball technique as well. Overall, 17 descendants, who were at various ages when their families were affected by the “Asanace” campaign and expulsion, were interviewed. The recruitment of participants and the interviewing were concluded after exhausting all possible avenues to reach “Asanace” campaign survivors and their families.

Data were collected using semi-structured in-depth interviews comprising three general topics with six optional open-ended questions focusing on memories of the “Asanace” campaign and the subsequent expulsion from the former Czechoslovakia in addition to their emigration experiences. The interviews inquired about family and relationships within the family, while also giving space to share their personal experiences of being a descendant of political dissidents and the influence it has on their lives more broadly.

Two qualified and trained (e.g., psychotherapy and qualitative methodology) psychologists (one female and one male) conducted the interviews. From the beginning, each participant was in contact with just one interviewer, which allowed for the establishment of a relationship. All participants were informed about the goals of the study, and they were introduced to the institution under which the study was conducted. The interviewers also briefly introduced themselves and their interest in the topic and were open to answering any questions participants might have. The interviewers had no personal or family experience with the “Asanace” campaign or any other forms of oppression in their families during the communist regime in Czechoslovakia.

Each interview lasted from 60 to 120 min. The interviews were conducted one-on-one during a face-to-face meeting or video-call with those participants living abroad. All interviews were audio-recorded and later transcribed verbatim. The interviews were analyzed in the language in which they were conducted, and snippets of these were translated into English for publication purposes.

### 2.2 Participants

These inclusion criteria were implemented to enhance homogeneity in the presented sample of individuals who went through the “Asanace” campaign and subsequent expulsion from the country while in a specific developmental stage (Smith and Osborn, 2008; Smith et al., 2009): (a) children of Czechoslovakian dissidents who were victims of the “Asanace” campaign, (b) emigration to another country because of the “Asanace” campaign, and (c) the beginning of primary school education in the year of emigration, i.e., ages 5 to 7 during emigration. The participants were selected according to these inclusion criteria from a larger pool of descendants of survivors of the “Asanace” campaign who participated in our multigenerational study.

Therefore, five individuals qualified and thus were included in the presented study, with the majority of interviews conducted in Czech in person, and one in English via an online video-call. This formed a relatively small yet carefully considered sample. The sample includes three men and two women (see Table 1 for sample characteristics). All participants were adults between 46 and 50 years of age (*M* = 48.4 years, SD = 1.52) at the time the interviews took place.

**Table 1:** Characteristics of the participants (names have been anonymized).

	Harry	Elliot	Bobby	Lia	Mathilda
Gender	Man	Man	Man	Woman	Woman
Age	50	49	49	48	46
Highest education	Master’s degree	High school with diploma	Bachelor’s degree	Master’s degree	Master’s degree
Marital status	Divorced	With partner	Married	Single	Married
Interview language	Czech	Czech	Czech	English	Czech
Year of expulsion	1976	1978	1981	1979	1981
Age in expulsion	5	6	7	5	7
Region of emigration	Western Europe	Central Europe	Northern Europe	North America	Central Europe
Current residency	Czechia and country of emigration	Country of emigration	Czechia and country of emigration	Country of emigration	Czechia

### 2.3 Ethical considerations

This study was conducted according to the guidelines of the Declaration of Helsinki. Ethical approval for this study was obtained from the local ethics committee of the National Institute of Mental Health, Czech Republic (approval numbers 88/20 and 94/21) on March 25, 2020, and March 31, 2021, respectively. All participants were informed about the goals and procedures of the study and all participants signed a written informed consent before participating in the study. All participants understood that all data will be deidentified to ensure their anonymity; that is why we opted to anonymize not only their names but also the countries to which they emigrated.

### 2.4 Data analysis

The verbatim interview transcripts were analyzed using interpretative phenomenological analysis (IPA). This is a qualitative methodology, informed by phenomenology and hermeneutics, and thus is developed for the examination of personal lived experience while emphasizing the meaning-making by both participant and researcher (Smith and Osborn, 2008; Smith et al., 2009). It consists of an idiographic detailed analysis of personal experience case-by-case, while looking for themes and then for patterns across cases (Smith, 2017). Our analyses proceeded as follows: each case was analyzed individually, experiential statements were formulated, then subsequently clustered. Personal experiential themes and subthemes were identified, then cross-case analysis was performed. Two authors conducted independent analyses of the interviews and then discussed emerging (sub)themes.

## 3 Results

The analyses revealed that participants’ experiences can be categorized into four main group experiential themes. These are: 1. *Creating boundaries between themselves and the dissident legacy of their parents, 2. Campaign “Asanace” means emigration, 3. Who am I?* and *4. Judicious lessons from family history*. See Table 2 for a summary of the four group experiential themes and subthemes, together with the number of participants contributing to each and their names.

**Table 2:** Summary of group experiential themes and subthemes.

Themes and subthemes		*n* of participants contributing and their names
Creating boundaries between themselves and the dissident legacy of their parents		
	Feeling proud	5 (All)
	Preserving the family stories and history	3 (Harry, Elliot, Mathilda)
	History of emigration and opposition against the system in previous generations	4 (Harry, Elliot, Lia, Mathilda)
	Fear of family history repeating itself	2 (Harry, Lia)
	Living in the shadow of their parents’ stories	5 (All)
	Owning one’s own life story	4 (Elliot, Bobby, Lia, Mathilda)
Campaign “Asanace” means emigration		
	Affective responses	5 (All)
	Quick adaptation	5 (All)
	Emigration brings family together	5 (All)
	Family as stable basis with open communication	3 (Harry, Elliot, Bobby)
	Feeling misunderstood	2 (Lia, Mathilda)
Who Am I?		
	Being on the move	5 (All)
	Lack of stability	5 (All)
	Home as a psychological entity	5 (All)
	Czechs living elsewhere	4 (Harry, Elliot, Bobby, Mathilda)
	Broadened horizons and ability to adapt quickly	5 (All)
	Relationship with Czechia	5 (All)
	Czechia idealized in family narratives	4 (Elliot, Bobby, Lia, Mathilda)
	Importance of language in self-perception	4 (Harry, Bobby, Lia, Mathilda)
	Feeling different, otherness	5 (All)
	Feeling ashamed	2 (Lia, Mathilda)
	Being different as part of self-image	4 (Harry, Elliot, Bobby, Lia)
Judicious lessons from family history		
	The importance of freedom	5 (All)
	Engaging in cultural and political life	4 (Harry, Bobby, Lia, Mathilda)
	Meaningful experiences over material possessions	3 (Elliot, Lia, Mathilda)
	Living in the moment	2 (Elliot, Mathilda)
	Courage and taking risks	5 (All)
	Pass values across generations	4 (Harry, Elliot, Bobby, Mathilda)

### 3.1 Theme 1: creating boundaries between themselves and the dissident legacy of their parents

#### 3.1.1 Subtheme: importance of family history

All participants expressed feeling proud of their parents and their dissident activities in former Czechoslovakia, especially the human rights dimension, as expressed in this snippet:

*Lia: They went against some things, so I feel very proud of that and I had a chance to witness an example first-hand.*

Moreover, three of them also stressed the importance and motivation to preserve the stories and family history for next generations:

*Elliot: I think it’s important to document the history for the children. I don’t know in what form, whether by photos or writing the stories. It’s just important to document it in a neutral, non-judgmental, and non-polarizing way. (…) It’s important to look at it from a distance, without any political views. That’s how it should be presented to children.*

Interestingly, in families of four participants, there had been a history of emigration and opposition against the system in previous generations. Participants then connected the dissident activities of their parents with family history and additional examples of their ancestors, who were affected by and involved in other quintessential historical events that occurred on Czech territory, such as being part of the Czechoslovakian Legion during World War I^1^, surviving the Holocaust, being deported during the expulsion of Germans from Czechoslovakia after World War II, or grandparents emigrating during the Warsaw Pact invasion of Czechoslovakia in 1968:

*Harry: So, that’s how I found out how it was with my family before. That it’s something that’s dragged on for a long time. And various tragedies happened in our family. One family branch is Jewish, it was displaced to Poland, and only one survived. In another branch, there are Sudeten Germans who were expelled to Germany, to Bavaria. There are different stories. I am such a mixture of Czechoslovak history. (…) They took everything from everyone. Some were in prison, some emigrated. That was the story that followed me, and especially my father. It’s a very powerful thing. They confiscated the properties of our families, but after 1989, they returned it. And in all this, there is still this belief that there’s some justice. It’s just a matter of living long enough to see it.*

*Mathilda: All my great-grandfathers were in prison and fought for the opposition. (…) So, the fact that my mother was in prison is just like 1 + 1.*

However, two of them also expressed fear of family history repeating itself and a determination to do what it takes to not to repeat it in their actions, which even led Lia to a decision to not have children:

*Harry: When the tanks appeared in Crimea and eastern Ukraine in 2014. (…) I thought to myself, that’s obvious, it’s going back to what it was. I was convinced it was time to emigrate. For me, it was very difficult to deal with (…). I’m going to repeat what happened to me. I can’t do it like this. So, I stayed here. (…) I think it’s important not to transmit the negative phenomena.*

*Lia: So, it’s interesting because I chose not to have children (…) I honestly felt that the situation we grew up in was too hard and I didn’t wanna repeat it.*

#### 3.1.2 Subtheme: owning the story

Although, as can be seen in previous snippets, the participants expressed profound pride, appreciation, and honor toward their parents and their actions, they also felt like they are living in the shadow of their parents’ story:

*Mathilda: We are the children of this generation that signed everything, that was imprisoned, that is in the books, signed Charter 77; they are dissidents, chartists, but who are we? Nothing. (…) Our history does not exist. There is only the history of our parents.*

And four of them (except Harry) expressed the desire to change the story of the family, to not live in the shadow of their parents and to create boundaries between their story and their parents. For example, Elliot stated that his life *“has nothing much to do with the ‘Asanace,’”* and Bobby opposed *“the responsibility to take over their work or inherit their project.”* They described the process of defining themselves in their own life story beyond the story of their parents:

*Lia: I don’t fully own the story, like, sometimes I do not feel like the story is mine because I was a kid, you know, like, it’s the story of my mom, the story of my dad. I’ve been able to rearrange it for myself, so that it has … I don’t want to be a victim to that story.*

### 3.2 Theme 2: campaign “Asanace” means emigration

Spontaneously, the participants did not reflect memories of oppression and persecution on their parents (e.g., repeated interrogation, being taken into custody, eavesdropping, home inspections, threats). It seems that due to their age at that time, they were sheltered from these events or simply remembered very little from these times. For example, Mathilda referred to home inspection stories that she does not remember herself, but that she knows from family stories, where they have taken on a form of humorous sketches.

However, all participants spoke with affective responses about emigration. They remembered that emigration and moving to another country was something unimaginable, and that only through the emotional reactions of their parents they knew something was going on:

*Elliot: I remember that we packed our things. I wasn’t stressed out at all, but I remember that we were on the train and my mom cried.*

*Bobby: For me, the moving was sort of a game, like an adventure. But it was hard for my parents. They argued a lot. They were nervous and my mother especially took it really hard.*

As Harry mentioned, moving to another country was something he did not understand as a child. Although the participants knew about the move to another country, due to their young age, they lacked the understanding of implications and consequences. The participants then described their shock and confusion after settling down in the new country. Very briskly, however, they also shared that they were surprised how quickly they managed to adapt to a new culture and learn a new language:

*Lia: And all of the sudden, we came to the country, and I was very unhappy that we went, very unhappy, nervous. But it is amazing like human really adapt and is resilient.*

*Harry: So, I didn’t start the first class in Czech school —I didn’t know a word. I didn’t know anything. It was horrible, absolutely horrible. But that’s a child’s miracle, that you learn quickly, nearly instantly. (…) After a while, only my name indicated that I was from somewhere else.*

#### 3.2.1 Subtheme: family bonds and communication

Bobby, Elliot, and Harry referred to their families as their stable basis and their emigration experience as something that welded the family members close to each other. They also felt like they could talk with their parents about the experience, which in their perception is a shared one:

*Bobby: The life was in the family, and the family worked well. It has been an important base.*

*Elliot: We are talking about it with my father from time to time. The fact that we, as a family, went through two regimes is something that welds the family together. The only stable thing is the family.*

Although Mathilda and Lia also mentioned that emigration can bring families together, and especially during the process of settling in a new country this did happen, their overall perception of family is more negative. They felt misunderstood by their parents as if their parents did not perceive or they underestimated the impact of emigration on their mental health and development in general. For example:

*Mathilda: In our family, it is torn apart incredibly. (…) In our parents, there’s not a shred of empathy that we could have experienced it completely differently. (…) In Germany, they call the generation of my parents as blind—they are so preoccupied by themselves that they don’t look outside and perceive the world only through themselves. And I see it’s true for my parents.*

#### 3.2.2 Subtheme: ambivalence

Overall, all participants expressed ambivalent feelings toward emigration. On the one hand, they appreciated that the experience has enriched them culturally and linguistically. Yet on the other hand, they felt that it was very difficult to adapt to the new country without other family members present and that they now feel dispersion in their identity:

*Harry: I’m convinced that it’s a misfortune for every person to live in a country where they weren’t born and have no family.*

*Mathilda: For a long time, I thought how great it was, emigrating. That it just enriched me, that I got to learn two languages, live in two countries. Now, I don’t think so anymore. It’s great that I speak these two languages, but the ambiguity is terrible.*

### 3.3 Theme 3: who am I?

All participants deal with identity dispersion that is connected to three main common themes: the internal identity challenges of uprooting, feeling stuck between two cultures, and fighting for normality. These themes are present regardless of the characteristics of family bonds and communication in family.

#### 3.3.1 Subtheme: uprooting

The analysis revealed that all participants are trying to find answers to the following questions: Where do I belong? Where is my home? They described not having deep roots in any particular place. Although this feeling can be linked to the ability to adapt quickly and to feel well in different places, it is also linked to the lack of stability or to feeling as though they are constantly missing something. In their life stories, being on the move is a re-occurring theme: they have moved a lot and now often seem to live in two countries simultaneously.

*Bobby: Of course, whatever country I live in, I’ll be missing something. And I’ll travel here and there. (…) But in my life, as I would like it, I would like to have such a solid background. Like a house with a garden, where I could have my things, such security. (…) Now, I have two homes and none.*

*Mathilda: I dream about it, but I think it’s a utopia, it’s never going to happen. I feel best when I can travel. I don’t feel well when I’m settled down. But I’d want it desperately. (…) There’s this contradiction. I want to settle down, but I don’t. I want it, but I don’t feel good about it.*

For the participants, home is then not tied to a particular physical location but rather it is a psychological entity that they carry within themselves, and a desire that they have, wherever they go or move. It is a place *“where I feel good, where I can hang my hat”* (Harry), or as Bobby described: “*it’s a feeling that you have something in common with people around you, not only language but the frame of reference.”* Elliot even described the feeling that he does not know what it is like to *“feel at home,”* while Lia feels *“at home in all the places,”* and Mathilda describes feeling at home *“on the train.”*

#### 3.3.2 Subtheme: stuck in-between two cultures

As previous excerpts suggest, the experience of uprooting is closely tied to the participants’ bi-cultural backgrounds and their use of multiple languages. It is also part of the ambivalence associated with emigration. Most of them (except Lia) identified themselves as Czechs who have lived in the new country for a very long time. They described themselves as being stuck between two cultures, not fitting into either of them:

*Harry: I got to know a culture that is different from the Czech one. It’s not always easy for me to live here [in Czechia], because I still have the other culture. I’m at the halfway mark.*

*Bobby: I’ve always felt like the new country is foreign to me. For example, I’ve never known any lullabies or how to talk to children because I’ve never experienced that. I don’t know it because I’ve never lived through it.*

Although this mainly concerns challenges in establishing national identity and building relationships with peers, Harry also illustrated this through a bureaucratic example, as he received a certificate of exemption from military duty in both countries:

*Harry: They didn’t like that I’m Czechoslovak, so they didn’t accept me and put me only in the reserve forces. (…) And when I was drafted in Czechoslovakia, I told them, “You know, I’m enrolled in the military forces of a NATO country, and you are the Warsaw Pact.” They didn’t know what to do with that. So, it’s all related to the emigration, stories like that. These are the hilarious stories from emigration.*

As navigating between the two diverse cultures was (and sometimes still is) challenging for them, nonetheless, they all appreciated the broadened horizons, the ability to have different perspectives and to be able to orient themselves in two different cultures and political systems:

*Elliot: Just the fact that I’ve experienced communism seems good to me because my colleagues here in [city of emigration] can’t imagine that there could be another political and economic system, and I can tell them, “Oh, I've experienced two.” And I don’t want to say which is better, just that it’s possible. It’s just a slightly broader perspective. (…) Due to the fact that I’ve always been in-between cultures, that I’ve never fit in, I’m good at orienting myself in culturally diverse circumstances or groups of people.*

*Bobby: Being a foreigner in a country gives you the opportunity to see its problems from the outside or the typical character of its society.*

The analysis revealed that how one can relate to the Czech Republic is an ever-occurring question for those who return to Czechia after emigration as well as for those who stayed abroad. Most of them felt that the family narratives regarding the Czech Republic were idealized, and this often created a desire to live, visit, or otherwise engage with Czechia:

*Elliot: When I was a child, we always talked about Prague. How beautiful it was, the old Prague. It was idealization, really.*

*Mathilda: Just because of that, me and my sister have any relationship to this country. Both me and my sister, we came back here just because our parents projected their nostalgia onto us; otherwise, we wouldn’t have come back.*

Moreover, Czech was a language spoken at home for all the participants, while the other language was reserved for interactions with the outside world. As a result, most of the participants reflected on the difference in self-perception based on the languages they speak. Consequently, this creates a dispersion in their perception of themselves almost as if their identity was dependent upon spoken language. Lia even described the first years of going to school as a performance, highlighting the tension between her home language and the language used in the outside world.

*Bobby: You feel like a little bit of a different person. The language gives you different mentalities, different understandings.*

*Mathilda: When I speak [the other language] I feel competent and organized. When I speak Czech, I feel inferior and lost. (…) But Czech language, I have so connected with emotions that when I speak with my children in [the other language], I feel disconnected, like I’m not expressing emotions like I should.*

#### 3.3.3 Subtheme: fighting for “normality”

All participants described difficulties fitting into the new culture that were associated with feelings of being different or feeling like outcasts; furthermore, they perceived themselves as an “other” due to their emigration experience.

*Elliot: I realized that I had to fight for normality all the time. (…) As a young person, you don’t want to be different.*

While attempting to fit into peer groups, both Lia and Mathilda even described periods when they felt ashamed of their family, especially their economic situation, the neighborhood in which they lived, and perceived differences between their families and those of their local friends:

*Lia: I would say I became ashamed and embarrassed of our situation, but also, we weren’t regular immigrants that I knew, like everyone that came from Jamaica. (…) I chose to hide it; I don’t even know how I was so aware of class.*

*Mathilda: It was a strange feeling that I don’t fit in, that I don’t speak the language, that I don’t know anything, I have the wrong clothes. I wasn’t one of them. I still got the feeling that I’m from the periphery; it was more interesting everywhere else, so I didn’t want to stay there.*

Most of the participants managed to incorporate their experiences of feeling different into their self-image, as Elliot states: *“[I] worked on my self-confidence, that it’s okay to be a foreigner, that’s what protects my identity.”* However, Mathilda still struggles with feeling like an outcast and often feels like fighting for her “normality.” This is especially prominent in the Czech environment. Overall, this seems to further reinforce her tendency to be constantly on the move:

*Mathilda: I’ve found out that I don’t belong here at all. I’m weird. I’m uprooted. They even told me so at work! (…) I have a secret plan; I want to move to [city]. My husband doesn’t even know that, but I’ll do it. I won’t even tell him until a week before. I just need to do it. He’s great, but he wouldn’t understand.*

### 3.4 Theme 4: judicious lessons from family history

Despite the ambiguous attitudes toward emigration and the impact of their childhood experiences on all the participants, all of them took judicious lessons from their family history and in relation to this they emphasized the importance of personal autonomy and the pursuit of meaningful experiences over material possessions or security. In general, they expressed how their familial past experiences have shaped their values and attitudes toward freedom and toward engaging in cultural and political life.

*Harry: It gave me an idea of how freedom should look like and awareness of why one should be free. And that it’s worth fighting for freedom.*

The participants defined living in freedom as the ability to choose where one lives, to live according to one’s values, and to have access to information and experiences; additionally, Mathilda namely mentioned her love of theater and Harry noted his enjoyment talking with miscellaneous people in pubs and books. Their responses expressed skepticism about looking too far into the future and instead emphasized the importance of living in the moment.

*Elliot: I’ve also never believed that one could build and preserve anything. The only thing that works is present moments and experiences. (…) I think that the future will consist in experiencing those moments.*

*Mathilda: I don’t put roots down because I’m afraid that (…) I’ll lose it. It may be healthy not to count on what you have or what is now.*

In this context, all participants discussed the importance of courage, taking risks, and standing up for one’s beliefs, or as Lia stated, *“being a fighter,”* even if it means being in opposition:

*Bobby: I’ve always stood by what I believe in and never hidden what I’m thinking. I’ve always been willing to sacrifice some comfort for some opinions. (…) So, my father was in opposition and I, without me seeking it, ended up there somehow as well.*

Moreover, those with children considered it essential to pass these values on to them. This highlights the continuity of the values associated with the “Asanace” campaign across generations. Not only have participants’ experiences of the actions of their parents and the historical events surrounding the “Asanace” campaign shaped their values and beliefs, but they also see them as valuable enough to pass on to the next generation:

*Mathilda: Courage. That’s what I’m trying to teach them. If they don’t like something, they should say it, they should stand their ground.*

*Harry: I'm trying to pass on [to his son] the essence - that he’s supposed to be free. He’s supposed to choose where he’s comfortable, whether here or elsewhere. Not to be afraid to move out of the apartment or country. To be interested in foreign countries, having an overview is very important.*

## 4 Discussion

In this study, we focused on previously unexplored subjective experiences of the 1.5 generation of Czechoslovakian dissidents. Specifically, through in-depth, semi-structured interviews, we examined the narratives of five adults who were children old enough to remember and to be psychologically affected by living through the oppressive actions of the State Security, which led to the expulsion of their family from the country, i.e., the “Asanace” campaign. Nonetheless, due to their age at that time, they lacked the mature understanding of what was happening to them or their families. Using interpretative phenomenological analysis (Smith et al., 2009), we identified four significant themes in their experiences that not only reflect the long-term impact of political oppression on individuals and their families but also underscore the lasting effects of emigration on the development of children in general. Furthermore, these themes highlight the complexities of identity formation, and the challenges of navigating historical legacies with the interplay between personal narratives and collective memory. This study is among the few that focus on exploring the long-term consequences of historical totalitarian trauma in Eastern and Central Europe, where “up to four generations have lived since these haunting events” (Maercker, 2023, 03).

We focused on a group of child survivors of communist state oppression directed at families of political dissidents. However, our participants had only limited memories of these events. Mainly, they reflected on the expulsion from their home country, their feelings of being uprooted and their challenges of enduring the abrupt change in their lifeline. As previous studies suggest, children are not able to tell a coherent and detailed narrative about their life experiences during their pre-school years, which is a crucial milestone in the development of autobiographical memory (Lagattuta and Wellman, 2001; Fivush, 2011). Thus, the participants’ recollections and interpretations of the “Asanace” campaign are influenced by their developmental stage at that time. Nonetheless, exposure to such potentially traumatic events during this sensitive developmental period can influence one’s sense of self, the development of coping strategies, attachment, and interpersonal relationships (Dunn et al., 2017; Fivush, 2019).

This raises a question: Could they fundamentally be classified as 1.5-generation migrants? Perhaps the answer is “yes” and “no.” They fulfill the common characteristics associated with 1.5-generation migrants: their involvement in the decision-making process was limited, a portion of their socialization occurred in the country and culture of origin, and they experienced feelings of displacement followed by a period of adjustment (Orellana et al., 2001; Bartley and Spoonley, 2008). Furthermore, common to 1.5-generation migrants, they described difficulties in acculturation, identity formation, struggles for belonging, biculturalism and bilingualism (Rumbaut, 2004; Kebede, 2010; Gindelsky, 2019). It could be argued that while their families were expelled from the country, the children themselves, due to their age, did not experience it much differently than other emigrant children with non-dissident backgrounds. However, the data indicated specific nuances with which descendants of Czechoslovakian dissidents were confronted, creating additional challenges they have had to overcome. As they grew older, they gained a deeper understanding of the oppression and their parents’ and family’s unique legacy. Furthermore, since then, they have faced (public) reflections on the dissident activities of their parents, which has seemingly also affected their current experience as descendants of dissidents.

Their parents (and their entire family) went through a very particular and distressing experience and had a specific position in the country of origin, first labeled as the “enemies of the state” and later recognized as “fighters for democracy” after the Velvet Revolution in 1989. This further impacted their negotiation of identities above and beyond the emigration experience. Most of them felt the need to delineate their life narratives from the historical context of their parents’ actions and legacies. While participants expressed pride and gleaned judicious lessons from their parents’ activism, fostering their proclivity to active citizenship, it concurrently created areas to be navigated within their relationships with their parents, including tension in some cases. Furthermore, it seems that it increased the urgency of the questions regarding their national identity and their connection to the country of origin, its history and politics, due to the historical legacy itself, yet particularly due to their parents’ idealized reminiscences.

Interestingly, laced with ambivalence was the profound sense of “in-betweenness” that we observed in our participants (Bartley and Spoonley, 2008, 68). They perceived themselves as being in-between cultures, in-between fluctuating identities, and living in-between amorphous notions of home, which presented unique challenges beyond those typically faced by children, as previously documented by Kebede (2010) in study of 1.5-generation Ethiopian refugees.

It has been suggested that the way parents discuss and reminisce about past experiences and life stories with their children can influence their coping abilities and mental well-being into adulthood (Fivush, 2011; Marshall and Reese, 2022). For example, maintaining silence about potentially-traumatic experiences within the family has been reported as a risk factor (Danieli, 1998). Within the families of our participants, we observed differences in family bonds and communication styles. In line with previous studies (Lichtman, 1984; Braga et al., 2012; Dalgaard et al., 2019), we found that those participants who felt heard and understood in their family and whose parents shared their own experiences as well as factual information, showed signs of better adjustment and stability later in their lives, than those for whom communication within their family was more stifled, indirect and fragmented, e.g., in a form of funny sketches.

Suleiman (2002) argued that similar patterns of attitudes, behaviors, and affects tend to emerge in people who experience similar trauma at similar ages. This phenomenon is denoted as generational consciousness, which, in the case of the Holocaust, took many years to develop, ultimately leading to the formation of self-conscious generational units as well.

In the case of descendants of Czechoslovakian dissidents, this process is in the beginning stages. Until now, their experiences were hidden in the shadow of their parents as publicly known figures throughout Czech society. As noted by Eyal (2004), these public images are skewed by the prevailing “will to memory” in Czech society to overcome the collective traumatic memory of the experience of communism via positioning dissidents as the “transcendent pastors of the individuals that compose civil society, whose consciences they guide” (Eyal, 2004, 8). Although these portrayals offer valuable and judicious lessons for children (and society as a whole), they do not provide a full picture of who their parents are (were) and the role they play(ed) in the lives of their children. Furthermore, there appears to be a need for descendants to distance themselves, to form their own perceptions of themselves and their family, and to separate from these public images, which suggest the importance of the interplay between personal, subjective narratives and collective memory.

## 5 Limitations and future studies

This is the first study focusing on the experiences of the descendants of dissidents; thus, this study presents the first glimpses of the ways they may interpret their life stories. Balancing the constraints given by the focused criteria and limited participant availability, Smith et al. (2009) assert that five interviews can be sufficient for interpretative phenomenological analysis. However, other authors (Hennink et al., 2017; Sebele-Mpofu, 2020; Hennink and Kaiser, 2022) have underscored the distinction between code and meaning saturation when considering sample size. While the homogeneity of our sample and the consistency and elaboration of themes across interviews indicate the achievement of a satisfactory level of code saturation within themes identified in our study, achieving a good level of meaning saturation, i.e., capturing subtle nuances within the themes, might have required a larger sample size. Nonetheless, the coherence, explicitness, and depth of the interviews enabled the identification of themes that outline the lived experiences of the 1.5-generation descendants of Czechoslovakian dissidents.

Future studies can build upon these insights to further explore and elaborate on the ways in which descendants of dissidents interpret their experiences, if possible, with a larger sample size that would enable achieving nuanced meaning saturation and increased understanding of the complexity (Hennink et al., 2017; Hennink and Kaiser, 2022). Furthermore, to enhance generalizability, exploring interpretations of the life stories of dissident descendants from other countries with differing regimes would be beneficial. Also, comparing their experiences with the experiences of 1.5-generation migrants who left their country voluntarily or were not expelled for political reasons could clarify the above-discussed differences and similarities between these groups. Moreover, focusing on the two remaining age sub-groups of 1.5-generation dissident descendants would broaden the understanding of developmental trajectories and how such historical events might impact individuals throughout the lifeline. Furthermore, an interesting avenue for future studies would be exploring the fulfillment of diagnostic criteria of (complex) post-traumatic stress disorder in samples of descendants of dissidents, which is beyond the scope of this current study.

## 6 Conclusion

The presented study delved into the previously unexplored experiences of descendants of Czechoslovakian dissidents, the so-called 1.5 generation. Through in-depth interviews, the study focused on the long-term psychological consequences of political oppression and expulsion on individuals and their families. The stories of the five now-adult participants highlight the interplay between subjective narratives and collective memory, as this generation has lived in the shadows of their parents and their parents’ political activities, which made them publicly known figures in both regimes, albeit with very different connotations.

## Data availability statement

The raw data supporting the conclusions of this article will be made available by the authors, without undue reservation.

## Ethics statement

The studies involving humans were approved by Ethics Committee of the National Institute of Mental Health, Czech Republic. The studies were conducted in accordance with the local legislation and institutional requirements. The participants provided their written informed consent to participate in this study. Written informed consent was obtained from the individual(s) for the publication of any potentially identifiable images or data included in this article.

## Author contributions

ND: Conceptualization, Data curation, Formal analysis, Investigation, Methodology, Project administration, Writing – original draft. RH: Data curation, Formal analysis, Investigation, Writing – review & editing. ES: Supervision, Writing – review & editing. MP: Conceptualization, Formal analysis, Funding acquisition, Methodology, Supervision, Writing – review & editing.

## Footnotes

^1^The Czechoslovak Legion comprised the armed forces that fought during and after World War I on the allied side in pursuit of independence from Austria-Hungary. It helped its leaders gain official recognition and international support for a political union of the Czechs and Slovaks and their national interests (Hauner et al., 2023).
